# Palpitations following regular ibuprofen dosing in a 13-year-old girl: a case report

**DOI:** 10.1186/1752-1947-4-76

**Published:** 2010-03-02

**Authors:** Robert J Douglas

**Affiliations:** 1 Seaton Medical and Specialist Centre, 175 Trimmer Parade, Seaton, South Australia, 5023, Australia

## Abstract

**Introduction:**

The sensation of palpitations may either be the initial or the only symptom of cardiac arrhythmia. We describe a case of an apparent clear temporal relationship between standard ibuprofen dosing and palpitations. A review of the medical literature revealed this to be, to the best of our knowledge, the first reported case of this type.

**Case presentation:**

A 13-year-old Caucasian girl initially presented to our clinic with hamstring tendinitis. She was commenced on a medication regimen of paracetamol and ibuprofen. After the third ibuprofen dose, she experienced palpitations. These were associated with lower chest and/or upper abdominal discomfort, and a feeling of being hot and sweaty. Her symptoms ceased upon the cessation of ibuprofen therapy.

**Conclusion:**

Cardiac arrhythmia is a potentially fatal disorder that may exhibit heart palpitations as its initial (or only) symptom. The prompt recognition of the cause of the symptom can reduce mortality and morbidity associated with any underlying pathological processes. There is a need to investigate cases of recurrent palpitations so as to exclude underlying structural cardiac pathology and/or abnormal cardiac rhythm.

## Introduction

Tendinitis, or the inflammation of a tendon, is a musculoskeletal disorder that is commonly seen in patients in general practice clinics. It is usually treated with a combination of rest, simple analgesics, a non-steroidal anti-inflammatory drug (NSAID) and physical rehabilitation. In this case report, we describe a case of cardiac palpitations that temporally appears to be caused by standard ibuprofen dosing, an event that has not been previously described in the medical literature.

## Case presentation

A 13-year-old Caucasian girl weighing 45 kg was examined in a general practice clinic with a short history of left posterior thigh pain. There was no obvious precipitant condition for her complaint. She reported no significant medical history, took no medications and had no allergies. There was also no significant family medical history. A clinical diagnosis of left hamstring tendinitis was made. She was commenced on a regimen of paracetamol 1 g qid prn, ibuprofen 400 mg tds, and a gentle exercise and stretching program.

Shortly after taking the third 400 mg dose of ibuprofen, she felt hot and sweaty, and reported a sensation of palpitations. There was associated lower chest and upper abdominal discomfort, but she felt no frank chest or abdominal pain. No other symptoms, in particularly those suggestive of either an allergic reaction or an acute coronary syndrome, were noted.

Our patient continued with ibuprofen treatment for another day, with exacerbation of her symptoms after each ibuprofen dose. After informing her mother of her symptoms, her ibuprofen medication was discontinued. Her symptoms ceased thereafter.

**Figure 1:**
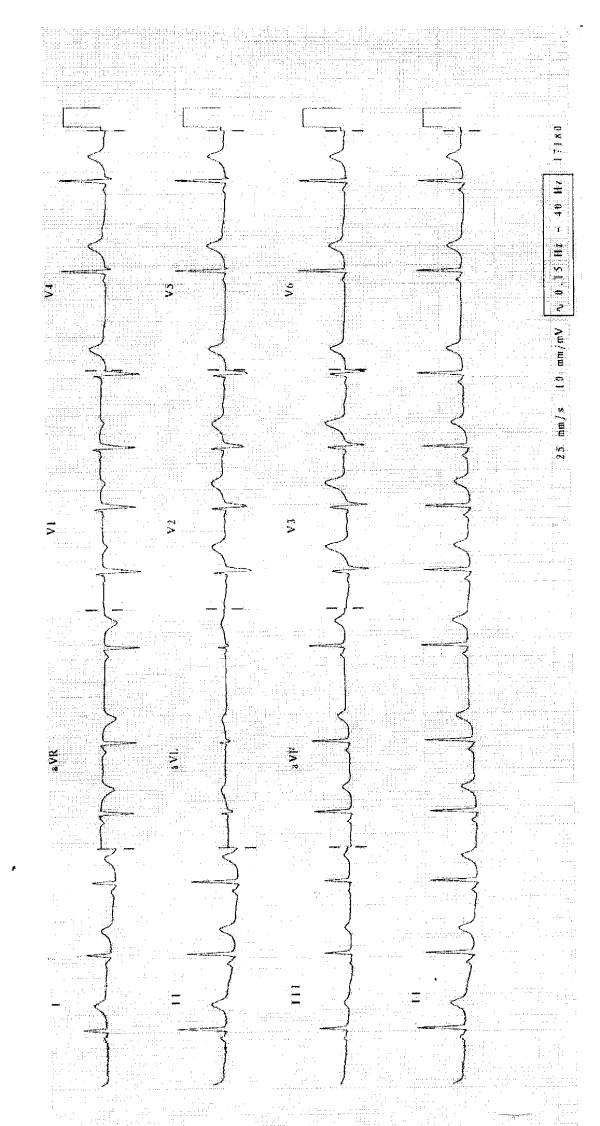
**An electrocardiogram performed after our patient's symptoms had resolved demonstrates sinus arrhythmia**.

Upon clinical review a few days later, she was found well with a pulse of 70. An electrocardiogram (ECG) test demonstrated sinus arrhythmia with no abnormality (Figure 1). Her echocardiography result was normal. Subsequent review by a pediatric cardiologist elicited no abnormality. Further review in the clinic determined that her symptoms had not recurred. After discussion with our patient and her mother, a decision was made to not rechallenge her with ibuprofen.

## Discussion

Ibuprofen was the first non-aspirin NSAID to be approved for over-the-counter (OTC) use, and is widely considered to be the best tolerated drug of its class [1]. Previous studies [2,3] have demonstrated that adverse drug reactions (ADR) to NSAIDs predominantly involve the skin, the gastrointestinal tract, and the respiratory system. Although it had been previously suggested that ibuprofen may be involved in the development of ventricular arrhythmia [4], only recently has ibuprofen been demonstrated to produce arrhythmia both *in vitro *and *in vivo *(in guinea pigs) [5]. This is achieved by a dose-dependent shortening of the cardiac action potential (AP), and shortening of the effective refractory period (ERP). This had resulted in a decrease of excitation propagation within the heart, which may provide substrate for an arrhythmogenic reentry circuit.

Cardiac arrhythmia secondary to ibuprofen overdose has been described in the medical literature. McCune and O'Brien reported the induction of atrial fibrillation (AF) in a previously-well 35-year-old man [6]. Meanwhile, Menzies *et al. *reported a case of broad complex tachycardia secondary to fulminant hyperkalaemia induced by ibuprofen overdose [7]. Interestingly, Gurfinkel *et al. *[8] have found that the intravenous administration of ibuprofen could immediately suppress arrhythmia during a cardiac electrical storm, but this work was conducted in the presence of decompensated cardiopathy, a condition not shared by the our patient. That our patient experienced only symptoms common to those associated with the sensation of palpitations, with no symptoms suggestive of an allergic aetiology, suggests that this case was not one of Kounis syndrome [9], which has recently been reported after routine ibuprofen use [10].

## Conclusion

Our patient has suffered no recurrence of her symptoms, and in the absence of ibuprofen rechallenge, it is probable that standard dose ibuprofen was the causative link with her symptoms of palpitations. No firm conclusion can be drawn as to the underlying pathophysiological mechanism of her symptoms. This case appears to be the first case report in the medical literature of an event adverse to cardiac arrhythmia following standard oral ibuprofen administration.

Cardiac arrhythmia is a potentially fatal disorder that may exhibit heart palpitations as its initial (or only) symptom. The recognition of drug-induced cardiac disturbance is necessary to prevent the progression of symptoms to a more serious adverse event, and to avoid reexposing patients to ibuprofen with resultant recurrent cardiac arrhythmia. Further research is required to identify the pathophysiological mechanism of ibuprofen-induced cardiac arrhythmia.

## Competing interests

The author declares that they have no competing interests.

## Consent

Written informed consent was obtained from the mother of our patient for publication of this case report and any accompanying images. A copy of the written consent is available for review by the Editor-in-Chief of this journal.

## References

[B1] Moore N Ibuprofen: a journey from prescription to over-the-counter use J R Soc Med 2007 100 Suppl 48 2 6 18335846 10.1177/014107680710004801s01

[B2] Leroy S Mosca A Landre-Peigne C Cosson MA Pons G Ibuprofen in childhood: evidence-based review of efficacy and safety Arch Pediatr 2007 14 5 477 484 10.1016/j.arcped.2007.01.012 17344039

[B3] Titchen T Cranswick N Beggs S Adverse drug reactions to nonsteroidal anti-inflammatory drugs, COX-2 inhibitors and paracetamol in a paediatric hospital Br J Clin Pharmacol 2005 59 6 718 723 10.1111/j.1365-2125.2005.02444.x 15948937 PMC1884871

[B4] Pratt CM Hertz RP Ellis BE Crowell SP Louv W Moyé L Risk of developing life-threatening ventricular arrhythmia associated with tefenadine in comparison with over-the-counter antihistamines, ibuprofen and clemastine Am J Cardiol 1994 73 5 346 352 10.1016/0002-9149(94)90006-X 8109548

[B5] Yang ZF Wang HW Zheng YQ Possible arrhythmogenic mechanism produced by ibuprofen Acta Pharmacol Sin 2008 29 4 421 429 10.1111/j.1745-7254.2008.00754.x 18358087

[B6] McCune KH O'Brien CJ Atrial fibrillation induced by ibuprofen overdose Postgrad Med J 1993 69 810 325 326 10.1136/pgmj.69.810.325-a 8321806 PMC2399662

[B7] Menzies DG Conn AG Williamson IJ Prescott LF Fulminant hyperkalaemia and multiple complications following ibuprofen overdose Med Toxicol Adverse Drug Exp 1989 4 6 468 471 2601619 10.1007/BF03259927

[B8] Gurfinkel EP Guerlloy FP Mautner B Suppression of life-threatening tachyarrhythmias, and atrio-ventricular ischemic block following the administration of anti-inflammatory intravenous drug Int J Cardiol 2007 114 2 E56 E57 10.1016/j.ijcard.2006.07.165 17067695

[B9] Kounis NG Zavras GM Histamine-induced coronary artery spasm: the concept of allergic angina Br J Clin Pract 1991 45 2 121 128 1793697

[B10] Kumar A Berko NS Gothwal R Tamarin F Jesmajian SS Kounis syndrome secondary to ibuprofen use Int J Cardiol. 2009 137 3 e79 e80 19482364 10.1016/j.ijcard.2009.04.049

